# Draft Genome Sequence of Aspergillus terreus High-Itaconic-Acid-Productivity Mutant TN-484

**DOI:** 10.1128/MRA.01170-19

**Published:** 2019-12-05

**Authors:** Shin Kanamasa, Tomoyuki Minami, Mitsuyasu Okabe, Enoch Y. Park, Tsukasa Fujimoto, Anna Takahashi, Masataka Murase, Shuichi Fukuyoshi, Akifumi Oda, Kenji Satou, Hiro Takahashi

**Affiliations:** a Graduate School of Bioscience and Biotechnology, Chubu University, Kasugai, Japan; b College of Bioscience and Biotechnology, Chubu University, Kasugai, Japan; c Aureo Co., Ltd., Tokyo, Japan; d Research Institute of Green Science and Technology, Shizuoka University, Shizuoka, Japan; e Graduate School of Integrated Science and Technology, Shizuoka University, Shizuoka, Japan; f Graduate School of Science and Technology, Shizuoka University, Shizuoka, Japan; g Graduate School of Horticulture, Chiba University, Matsudo, Japan; h Faculty of Information Technologies and Control, Belarusian State University of Informatics and Radio Electronics, Minsk, Belarus; i Graduate School of Medical Sciences, Kanazawa University, Kanazawa, Japan; j Institute of Medical, Pharmaceutical, and Health Sciences, Kanazawa University, Kanazawa, Japan; k Faculty of Pharmacy, Meijo University, Nagoya, Japan; l Faculty of Biological Science and Technology, Institute of Science and Engineering, Kanazawa University, Kanazawa, Japan; m Fundamental Innovative Oncology Core Center, National Cancer Center, Tokyo, Japan; University of California, Riverside

## Abstract

Itaconic acid is an important organic acid used in the chemical industry. Aspergillus terreus strain TN-484 is a high-itaconic-acid-productivity mutant derived from strain IFO6365. Here, we report the draft genome sequence of strain TN-484, advancing the understanding of the biosynthesis of itaconic acid in filamentous fungi.

## ANNOUNCEMENT

Itaconic acid is an unsaturated dibasic acid that is widely used as a raw material, food additive, and industrial product additive in synthetic resins and printing inks. Itaconic acid is known to be produced by filamentous fungi such as Aspergillus itaconicus and yeasts such as Candida tsukubaensis. Aspergillus terreus is used for industrial production of itaconic acid due to its high compound production (1). Itaconic acid is a relatively expensive organic acid, and further application expansion can occur with cost decreases (2). To more efficiently produce itaconic acid, we developed an itaconic acid-resistant strain, TN-484, by treating A. terreus IFO6365 with *N*-methyl-*N′*-nitro-*N*-nitrosoguanidine (NTG) and screened for an itaconic acid-resistant and high-itaconic-acid-productivity mutant (3). In addition, we cloned and analyzed the CAD1 gene encoding *cis*-aconitic acid decarboxylase (CAD). We showed that A. terreus CAD produced itaconic acid using *cis*-aconitic acid as the substrate (4). We previously showed that itaconic acid productivity was affected by medium pH and metal ion concentration in A. terreus (reviewed in reference 5). However, many itaconic acid biological functions remain unclear, and elucidating its underlying biosynthetic control will help clarify its functions.

TN-484 cells cultured in potato dextrose broth were homogenized using a bead crusher, and genomic DNA was isolated using a NucleoSpin plant II kit (TaKaRa Bio, Inc.). TN-484 genomic DNA was isolated from a liquid-submerged culture grown in potato dextrose broth at 30°C. Using the NEBNext Ultra II FS DNA library prep kit for Illumina (New England BioLabs [NEB], MA, USA), a 500-ng DNA aliquot was fragmented using DNA Fragmentase to generate DNA fragments (average, 600 bp) for library preparation. The DNA was end repaired and A tailed, adaptors were ligated, PCR was performed, and library purification was done according to the manufacturer’s instructions. Sequencing of the TruSeq DNA library (paired-end 2 × 300-bp reads) generated 18,647,110 reads. Removal of the sequencing primers and low-quality read trimming were conducted using CLC Genomics Workbench version 12.0.2 (Qiagen, Valencia, CA, USA), with default parameters. *De novo* assembly was performed using CLC Genomics Workbench, with default parameters. After *de novo* assembly, the reference-guided scaffolding of draft genomes was conducted using RaGOO version 1.1 (6), with default parameters, and A. terreus NIH2624 genome sequences (GenBank accession number GCA_000149615). The resulting genome assembly had a length of 29,733,777 bp divided into 24 contigs. The *N*_50_ contig length was 1,952,528 bp, the GC content was 52.5%, and the genome coverage was 174.9×. The coding regions of the chromosomes were predicted using AUGUSTUS version 3.2.2 (7) and implemented in OmicsBox version 1.1.164 (Qiagen), with default parameters, using A. terreus as a gene model (the A. terreus model is contained in the AUGUSTUS package). The estimated number of genes in the draft genome was 10,301. This genomic information could provide insight into the genetic basis of itaconic acid production by this strain.

### Data availability.

The draft genome sequence for strain TN-484 has been deposited in GenBank/ENA/DDBJ under the accession number BKZM00000000 (BKZM02000001 to BKZM02000024). The SRA/DRA/ERA accession number is DRA008975.
